# Early intervention in psychosis team (EIT): pathways to care

**DOI:** 10.1192/bjo.2021.322

**Published:** 2021-06-18

**Authors:** Chloe Uffendell, John Stevens

**Affiliations:** MerseyCare NHS Trust

## Abstract

**Aims:**

The main aim of this study was to investigate whether the EIT access and waiting time standard (>60% of people experiencing first episode psychosis (FEP) are treated with a NICE-approved care package within two weeks of referral) was being met within Liverpool EIT.

We also wanted to understand the pathway to treatment within EIT services, identify delays in the process of triage/assessment/MDT/medical review and implement changes to reduce delays.

**Method:**

This study was a retrospective cross-sectional audit of all patients accepted on to the FEP pathway following MDT discussion in the Liverpool EIT Teams across May and June 2020.

Case notes were analysed for delays in referral, engagement with assessment and care-coordinators, as well as prescriber review offering medication. The data were collated and analysed before implementing changes.

**Result:**

40 patients presented as FEP in May and June 2020, 6 were excluded due to an extended inpatient stay.

Within the remaining patient cohort (n = 34), 64.7% of patients were engaged with a care package within 14 days. Only 14.7% of patients received an offer of medication within 14 days, the mean time to be offered medication was 39 days.

26% of patients first contact within MerseyCare Trust was with EIT, 74% presented elsewhere. 24% instead presented to liaison psychiatry from A&E departments, 18% to the single point of access team, 9% to criminal justice liaison team (CJLT) and 9% to North West Ambulance Service triage car.

29% of referrals came from the community (GP and counselling services), 15% from CRHT (crisis resolution and home treatment team), 14% from CJLT, 12% from urgent care team, 9% from liaison psychiatry.

**Conclusion:**

The Access and Waiting time standard was met. However, this study showed that patients were not being referred to EIT at first point of contact. This study shows 26% of service users first presented to liaison psychiatry, yet only 1/3 of those were immediately referred to EIT, the remainder being later referred by other services e.g. CRHT.

In addition to referral delays, lack of medical practitioner availability caused significant delays in arranging medical reviews, delaying patients access to medication.

The changes implemented to address these issues included educating MerseyCare services in the early recognition of psychosis to increase early referral. Non-medical prescribers’ roles were developed to perform initial medical reviews in addition to doctors, allowing patients earlier medication access. This allowed ‘urgent slots’ to be developed, time set aside for emergencies enabling prompt review of urgent cases.

